# An Artificial Intelligence-Assisted Flexible and Wearable Mechanoluminescent Strain Sensor System

**DOI:** 10.1007/s40820-024-01572-5

**Published:** 2024-11-15

**Authors:** Yan Dong, Wenzheng An, Zihu Wang, Dongzhi Zhang

**Affiliations:** College of Control Science and Engineering, China University of Petroleum (East China), Qingdao, 266580 People’s Republic of China

**Keywords:** Mechanoluminescent, Strain sensor, Flexible, Deep learning, Wireless

## Abstract

The complex wiring, bulky data collection devices, and difficulty in fast and on-site data interpretation significantly limit the practical application of flexible strain sensors as wearable devices. To tackle these challenges, this work develops an artificial intelligence-assisted, wireless, flexible, and wearable mechanoluminescent strain sensor system (AIFWMLS) by integration of deep learning neural network-based color data processing system (CDPS) with a sandwich-structured flexible mechanoluminescent sensor (SFLC) film. The SFLC film shows remarkable and robust mechanoluminescent performance with a simple structure for easy fabrication. The CDPS system can rapidly and accurately extract and interpret the color of the SFLC film to strain values with auto-correction of errors caused by the varying color temperature, which significantly improves the accuracy of the predicted strain. A smart glove mechanoluminescent sensor system demonstrates the great potential of the AIFWMLS system in human gesture recognition. Moreover, the versatile SFLC film can also serve as a encryption device. The integration of deep learning neural network-based artificial intelligence and SFLC film provides a promising strategy to break the “color to strain value” bottleneck that hinders the practical application of flexible colorimetric strain sensors, which could promote the development of wearable and flexible strain sensors from laboratory research to consumer markets.

**Supplementary Information:**

The online version contains supplementary material available at 10.1007/s40820-024-01572-5.

## Highlights

- The sandwich-structured flexible mechanoluminescent sensor (SFLC) film shows great application potential as wireless wearable strain sensor and encryption device.
- System-level integration of SFLC film with deep learning-based artificial intelligence enables fast and accurate interpretation of color data to strain values with automatic correction of errors caused by varying color temperatures.
- The smart glove wearable sensor based on the SFLC film combined with deep learning neural network enables fast and accurate hand gesture recognition.

**Supplementary Information:**

The online version contains supplementary material available at 10.1007/s40820-024-01572-5.

## Graphical abstract

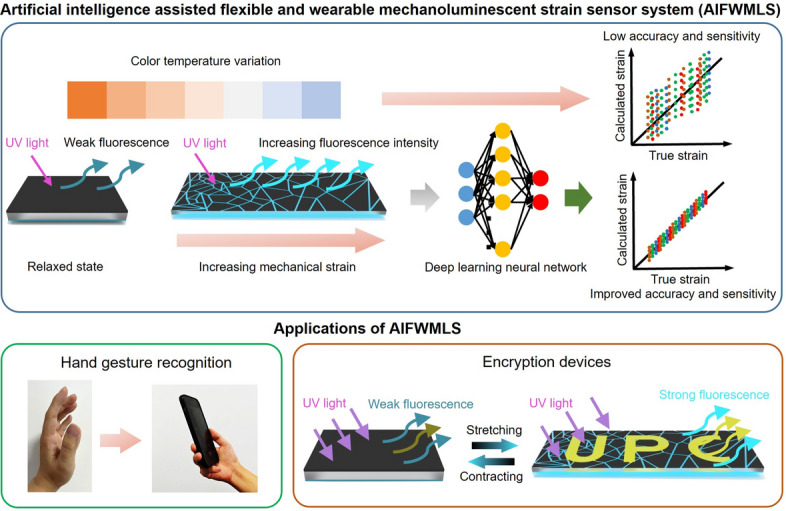

## Introduction

Wearable and flexible strain sensors are essential building blocks for the future Internet-of-Things, with its huge application potentials in the fields of health monitoring [1–3], human motion detection [4, 5], monitoring of mechanical deformation of core engineering materials [6], human–machine interfaces [7–10], and soft robotics [11, 12]. Most of the current wearable and flexible strain sensors convert mechanical deformation into electrical signals such as currents and voltages. This type of wearable sensors usually connects with bulky devices through complex wiring for data collection and processing, causing high power consumption, delays in reading of signal, limited working range, and great discomfort and inconvenience for users, which significantly hinders the practical application of this type of wearable strain sensors. Wireless wearable strain sensors transmit the generated electrical signal wirelessly through technologies such as Bluetooth [13, 14] and near-field-communication (NFC) [15, 16]. However, the integration of wearable strain sensors with the complex wireless unit and on-chip power-supplying unit significantly increases the size and rigidity of the wearable sensor, making the sensor electronics more vulnerable to deformations when attached to the soft human skin, which may compromise the credibility of the collected sensor data. The lack of system-level integration of high-performance strain sensitive materials with data collection and processing system poses a great challenge to the practical application of the wearable and flexible strain sensors.

Compared to conventional wearable strain sensors, flexible mechanochromic (MC)/mechanoluminescent (ML) strain sensors convert deformations to color signals, enabling the direct visualization of stress/strains [17–24]. Smart mobile devices or detectors such as smart phones can directly capture the colors induced by deformations through their cameras. Therefore, flexible MC/ML strain sensors do not need complex wiring or wireless communication units to transmit signals to data collection and processing devices, which could significantly increase the number of application scenarios of the sensor. The battery-free, wireless structure of the MC/ML strain sensor lowers the difficulty of sensor fabrication and cost. Self-developed and user-friendly smart phone apps or user interfaces integrated with powerful deep learning algorithm can boost color data processing and interpretation for further use such as human gesture recognition [25, 26]. However, to the best of our knowledge, such kind of wearable smart MC/ML system with automatic color data collection and interpretation has not been developed yet.

To improve the performance of the MC/ML strain sensor, tremendous efforts have been made in the research of highly sensitive MC/ML materials. For example, considerable progress has been made in developing high-performance photonic crystals with periodic microstructures [27–32], novel mechanophores [19, 33, 34], cholesteric liquid crystal elastomers (CLCE) [35, 36], centrosymmetric crystals [37], strontium-aluminate-based materials [38], and inorganic–organic composites with large difference in triboelectric series [39], as highly sensitive materials for MC/ML strain sensor. To improve the flexibility and wearability of MC/ML strain sensors, MC/ML strain-sensitive materials were composited with flexible elastomer fibers [40, 41] or coated on flexible fibers for potential use as wearable ML strain-sensitive textile [42]. And self-healing flexible MC/ML strain-sensitive materials were also developed to further improve wearability [43]. To further enhance the performance of the MC/ML strain sensor, dual-model MC/ML strain sensors were developed to realize multidimensional stress/strain sensing with both optical and electrical signals [24, 44, 45]. Despite the progress made in developing high-performance MC/ML strain-sensitive materials, MC/ML sensor color data acquisition, processing, and interpretation system and system level, seamless integration of the high-performance MC/ML strain-sensitive materials with the color data processing system is still lacking [20, 46]. For existing MC/ML strain sensor, expensive and bulky devices such as spectroradiometers or DLSR cameras capture the color data. Then, a separate system converts colors to strain values through complex and obscure process such as converting color signals to coordinates in the CIE 1931 color space diagram [17], making direct readout of strains difficult, especially for subtle color changes caused by small strains. The “color to strain value” bottleneck mentioned above poses a huge challenge to advance wearable MC/ML strain sensors from research laboratories to consumer markets.

To address the problems mentioned above, this work developed an artificial intelligence-assisted flexible and wearable mechanoluminescent sensor system (AIFWMLS). The AIFWMLS system is composed of a self-developed flexible mechanoluminescent sensor film, a deep learning neural network-based color data processing system embedded in a cloud server, and a user-friendly webpage user interface for bridging the sensor film and the data processing system. Firstly, we developed a sandwich-structured flexible mechanoluminescent sensor film (SFLC) through layer-by-layer assembly and lift-casting method, with hydrophobic and highly stretchable polydimethylsiloxane (PDMS) chosen as the middle layer elastic support. The SFLC film contains ZnS:Cu as fluorescent material in the bottom silica gel (SG) layer which emits fluorescence under UV light irradiation. A layer of CNTs on top of PDMS shields the fluorescence emitted from the bottom layer at the relaxed state. When the SFLC film is under strain, the fluorescence intensity of ZnS:Cu increases due to its intrinsic mechanoluminescent property. Moreover, the CNTs layer cracks and detectable fluorescence from the underlying SG-ZnS:Cu fluorescent layer become even stronger due to the gaps and slits generated by the reversible cracking of CNTs top layer. As a result, the intensity of the detectable fluorescence significantly increases with the increasing strain, which sets the foundation for strain detection using SFLC film. The developed strain-sensitive SFLC film is flexible, waterproof, and highly strain-sensitive, which can also serve as potential encryption devices. Taking advantage of the powerful deep learning-based artificial intelligence, a color data acquisition and processing system with deep learning algorithm captures color data from the SFLC film induced by strain and rapidly interprets collected color data to strain values. To address the errors in the predicted strain values caused by the varying color temperature in different measurements, for the first time, the developed color data acquisition and processing system with deep learning algorithm automatically corrects errors caused by varying color temperature, which significantly improves the accuracy of the predicted strains. Based on the AIFWMLS system, we developed a smart glove sensor array composed of several SFLC sensor films, which is capable of recognizing different hand gestures with the assistance of a user-friendly, cloud-server-based data acquisition and processing system developed with deep learning algorithm that can be operated on a smart phone. The data collection and processing system quickly captures the colors from the SFLC films on the smart glove and uploads the color data to a cloud server where the color data are processed by a trained deep learning neural network, enabling recognition of hand gestures. Compared to the visual-based system which uses very complex algorithm for feature extraction and complicated neural network model for gesture recognition, the AIFWMLS system developed in this study can do hand gesture recognition by only using the color data from the SFLC films. The features of color data are easy to extract, and the neural network model for hand gesture recognition is much simpler than that of the visual-based system, making hand gesture recognition much easier and faster compared to the visual-based system. Moreover, the SFLC film can also serve as a potential encryption device, demonstrating its versatility. The proof-of-concept, system-level integration of wearable and flexible mechanoluminescent sensors with deep learning neural network-assisted data collection and processing system provides a promising strategy to facilitate the practical application of wearable strain sensors by enabling facile, on-site sensor data collection and interpretation, which promotes the development of wearable and flexible strain sensors from laboratory research to consumer markets.

## Experimental Section

### Preparation of the Sandwich-Structured Flexible Mechanoluminescent Film

The SFLC film was fabricated through layer-by-layer assembly and lift-cast coating of the top CNTs layer. As shown in Fig. S1, firstly, PDMS base and curing agent were mixed and stirred for 15 min with a mass ratio of 15:1. Then, the mixture was degassed in vacuum and poured into a PMMA mold with a rectangular groove (length: 40 mm, width: 30 mm, height: 1 mm). A PDMS film formed after curing for 2 h under 60 °C. Silica gel (5-degree hardness) part A and part B were mixed with a mass ratio of 1:1 and stirred for 15 min. Then, ZnS:Cu phosphor powder and silica gel were mixed with a mass ratio of 1:10 and stirred for another 15 min to form the precursor for ZnS:Cu-SG layer. After degassed in vacuum, the liquid ZnS:Cu-SG precursor was poured onto the PDMS film in the PMMA mold and dried at 60 °C for 1 h. Then, the formed ZnS:Cu-SG/PDMS film was peeled off from the mold and attached to a glass slide with the PDMS layer facing outward. The top CNTs layer was coated onto the ZnS:Cu-SG/PDMS film through a “lift-cast” coating method. Specifically, 0.3 g of multi-walled carbon nanotubes (CNTs) were dispersed in 200 mL of anhydrous ethanol and ultrasonicated for 6 h until the CNTs were uniformly dispersed in the anhydrous ethanol. Then, the CNTs dispersion was sprayed onto the water surface using a spray bottle. CNTs uniformly distributed onto the water surface due to the interfacial tension. Next, a nanosponge was inserted into the water at the edge of the container, disrupting the equilibrium of the surface tension and causing the compression of the loose CNTs layer into a dense CNTs layer on the water surface. Then, the ZnS:Cu-SG/PDMS film was carefully inserted into the water from the edge of the contained free of surface CNTs layer. Then, the ZnS:Cu-SG/PDMS film was lifted from the water where the water surface was covered by CNTs. Since PDMS is a hydrophobic polymer, the surface of the PDMS layer is hydrophobic. Therefore, when the hydrophobic CNTs on the water surface come into contact with the hydrophobic PDMS surface of the ZnS:Cu-SG/PDMS film, CNTs automatically bonded to the PDMS surface, forming a CNTs layer. The “lift-cast” coating process was repeated several times. Then, drying the CNTs coated film at 60 °C under vacuum completed the fabrication of SFLC film. The fabrication method of the SFLC film with commercial fluorescent powder as fluorescent materials is the same. The only difference is that we substituted ZnS:Cu with commercial fluorescent powder and mixed it with SG in a mass ratio of 1:10 to form the fluorescent layer.

### Characterization of the SFLC Film

Scanning electron microscopy (SEM, Hitachi S-4800) was used to study the microstructure of the SFLC film. Photoluminescence spectroscopy (FLS1000) was used to characterize the intensity of detectable fluorescence of the SFLC film at different strain levels.

## Results and Discussion

### Design Principle of the AIFWMLS System

In most cases, conventional wearable strain sensor connects to bulky electronic devices for signal collection through complex wiring, which dramatically limits the working range of the wearable sensor. The signals collected from the sensor are often resistances or currents, which still need further interpretation to convert the collected electrical signals to strain values. The problems mentioned above significantly hinder the practical application of flexible and wearable strain sensors. Compared to conventional strain sensors, color signals of MC/ML strain sensors can be collected wirelessly through cameras on smart mobile devices, which facilitates fast and on-site color data interpretation to strain values and data display, making strain sensors more user-friendly and commercializable. However, most of the state-of-the-art MC/ML strain sensors lack such system-level integration of the highly sensitive MC/ML materials with color data collection and interpretation system, making on-site interpretation of color data to strain values difficult, especially for subtle color changes caused by small strains, which is difficult to detect and quantify. Moreover, the changing lighting conditions could significantly affect the collected color data and the predicted strains from the collected color signals, which could substantially deviate the predicted strain values from the true strain values.

**Fig. 1:**
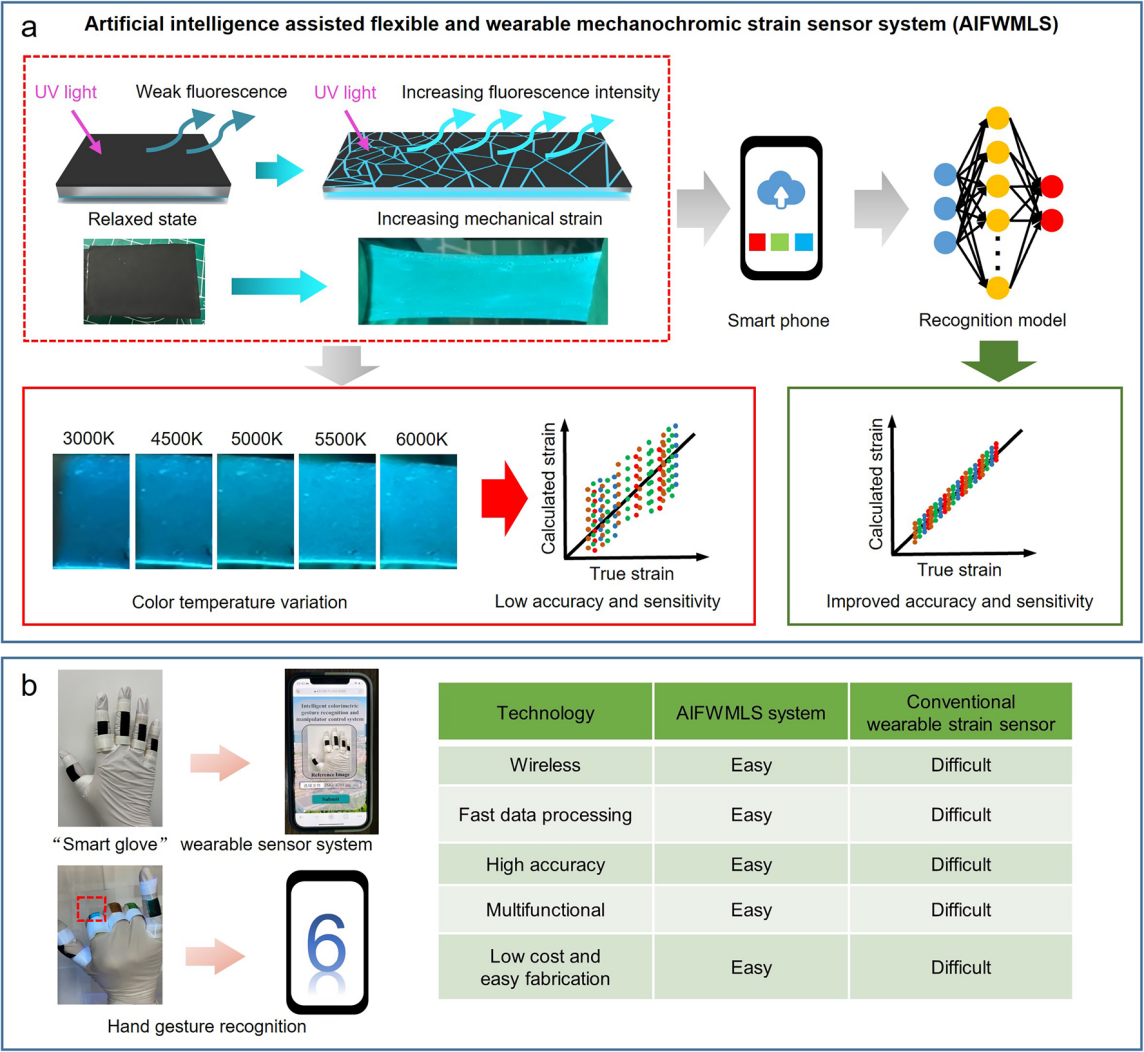
The concept of the artificial intelligence-assisted flexible and wearable mechanoluminescent sensor system (AIFWMLS) for strain sensing. **a** Schematic illustration showing the basic mechanism of AIFWMLS for strain sensing, the deep learning neural network can rapidly interpret colors to strain values with significantly improved accuracy under different color temperatures. **b** Schematic illustration showing the AIFWMLS system for hand gesture recognition, and comparison of the AIFWMLS system with the conventional wearable strain sensor

To address the problems mentioned above that hinders the practical applications of MC/ML strain sensors as wearable devices, this work developed an artificial intelligence-assisted flexible and wearable mechanoluminescent sensor system (AIFWMLS) (Fig. 1a). The AIFWMLS system integrates a sandwich-structured, flexible mechanoluminescent strain sensing film (SFLC) with a color data collection and processing system based on deep learning neural network. The bottom layer of SFLC film contains fluorescent material (ZnS:Cu) in silica gel (SG) which emits fluorescence under UV light irradiation. In the relaxed state, the film shows very weak fluorescence due to the shielding effect of the top CNTs layer. When the film is under strain, the fluorescence intensity of ZnS:Cu increases due to its intrinsic mechanoluminescent property. Moreover, the top CNTs layer cracks and detectable fluorescence from the underlying SG-ZnS:Cu fluorescent layer become even stronger due to the slits generated by the reversible cracking of CNTs top layer. As a result, the intensity of the detectable fluorescence significantly increases with the increasing strain. The data processing system in AIFWMLS, which is based on convolutional recurrent (CNN-GRU) deep learning neural network, collects the color data and interprets the color data into strain values with auto-correction of the errors caused by varying color temperatures in different measurements, which significantly increases the accuracy of the predicted strain values. With the assistance of the deep learning algorithm, the AIFWMLS system could predict subtle strain changes from color data, which increases the sensitivity of the strain sensor. To show the application potential of the AIFWMLS system, this work developed a proof-of-concept smart glove ML sensor array for hand gesture recognition (Fig. 1b). Compared to conventional wearable strain sensors, the AIFWMLS system can detect strains wirelessly with high accuracy and sensitivity through fast data collection and interpretation. Furthermore, the low-cost SFLC film in this work also shows the potential to serve as encryption device by encoding information in the SG-ZnS:Cu fluorescent layer, demonstrating the versatility of the SFLC film.

### Development of the Sandwich-Structured Flexible Mechanoluminescent Film

**Fig. 2:**
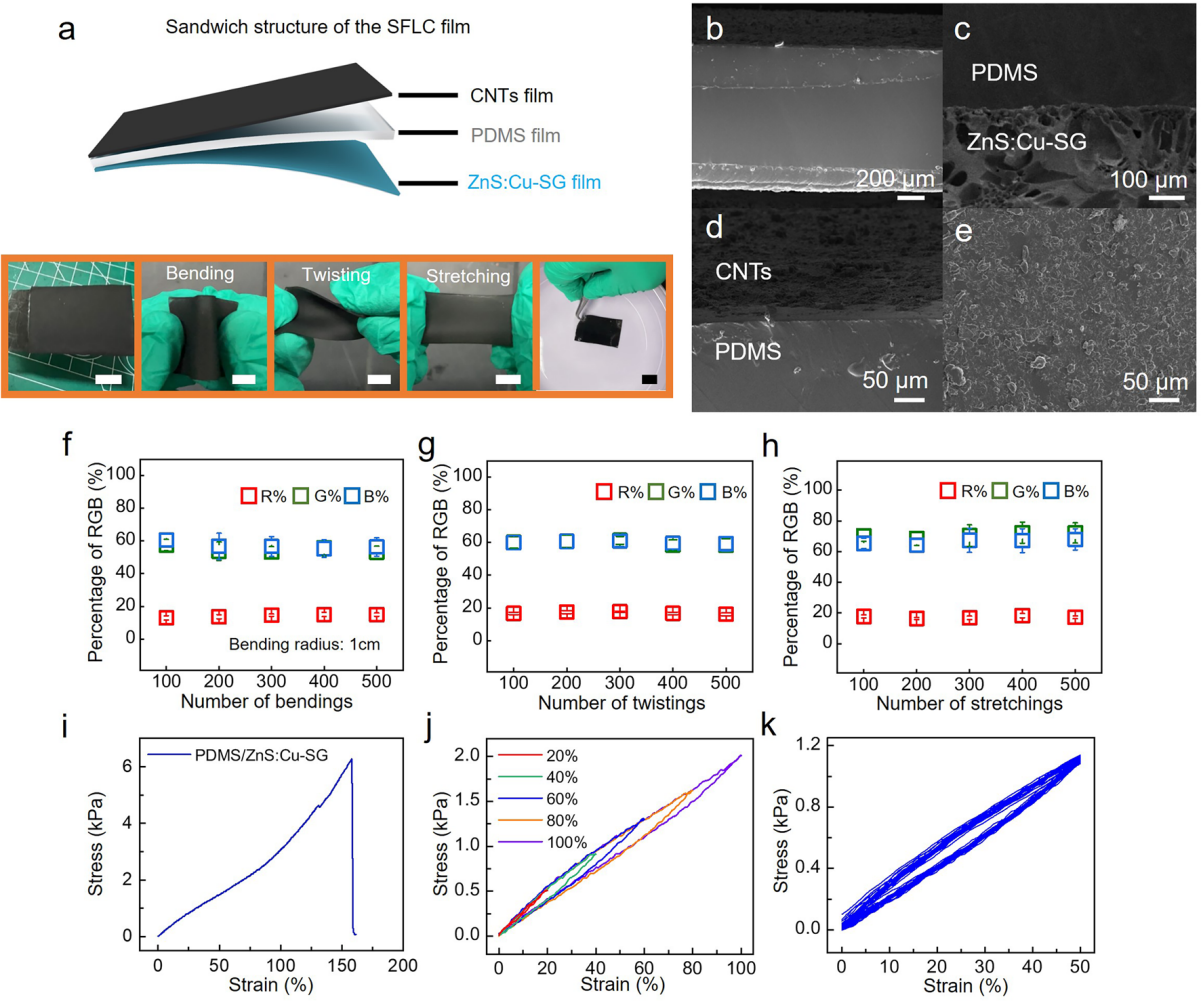
Structure and mechanical properties of the SFLC film. **a** Schematic illustration showing the sandwich structure of the SFLC film and photos showing the sensor film in bending, twisting, and stretching state (scale bar: 1 cm). SEM image showing the **b** sandwich structure of the SFLC film, **c** the interface between the PDMS and ZnS:Cu-SG layers, **d** the CNTs on PDMS layer, and **e** the morphology of the top CNTs film under stretch. Change of RGB (%) after numbers of **f** bendings, **g** twistings, and **h** stretchings of the SFLC film (n = 3, mean ± s.d.). **i-k** Mechanical properties of the SFLC film showing **i** the tensile stress–strain curve of the ZnS:Cu-SG/ PDMS film, **j** loading/unloading curves of SFLC film within 20% to 100% strain range, and **k** loading/unloading curves of the SFLC film at 50% strain

The layer-by-layer assembly of the SFLC film and lift-cast coating of the top CNT layer (details shown in experimental section and Fig. S1) yields a stable and robust sandwich layer structure, as shown in Fig. 2a. The SFLC film is composed of a bottom layer (ZnS:Cu-SG) of silica gel which contains fluorescent ZnS:Cu material (SEM images and XRD shown in Fig. S2), a top fluorescence-shielding layer of CNTs, and a layer of elastomer (PDMS) in the middle. Figure 2b shows the SEM images of the three-layer sandwich structure of the SFLC film. The thickness of the bottom fluorescent ZnS:Cu-SG layer is around 200 µm. And the porous ZnS:Cu-SG layer interfaces with the dense PDMS layer seamlessly with a well-defined interface (Fig. 2c). The seamless bonding between PDMS and fluorescent ZnS:Cu-SG layer renders strong adhesion between the PDMS layer and the ZnS:Cu-SG layer. The stripping test of the bonded ZnS:Cu-SG/PDMS layers (Fig. S3) shows that the largest stripping force between the bonded ZnS:Cu-SG/PDMS layers is about 550 N m^−1^, demonstrating the strong adhesion between the ZnS:Cu-SG/PDMS layers in the SFLC film. Figure S4a shows the stripping force versus displacement curves of the SFLC film after different stretching cycles. The stripping force at the beginning of detachment of the PDMS layer and the SG layer of the SFLC film did not change significantly after hundreds of stretching cycles (Fig. S4b), all within 540–550 N m^−1^, demonstrating the firm adhesion between the PDMS layer and the SG layer and the stable structure of the SFLC film. The small fluctuation of stripping forces of different SFLC films could be due to the individual difference of the five SFLC films used for the tests. Figure 2d shows the side view of a thin black CNTs layer uniformly distributed on top of the PDMS layer. The top view of the SFLC film under stretching (Fig. 2e) shows that CNTs did not come off from the deformed PDMS layer, and remained a uniform morphology, demonstrating the stable bonding between the CNTs layer and the PDMS layer produced by the lift-cast coating method. Other fabrication methods such as drop-casting method and brush-casting method did not result in uniform CNTs layers on top of PDMS (Fig. S5). The PDMS elastomer middle layer improves the mechanical properties of the SFLC film. The flexible, waterproof SFLC film can tolerate repetitive bending, twisting, and stretching (Fig. 2a). The percentage R, G, B values only exhibit negligible changes (≤ 5%) after bending (Fig. 2f), twisting (Fig. 2g), or stretching (Fig. 2h) for up to 500 cycles, demonstrating the robustness of the SFLC sensor film under repetitive deformation. As shown in Fig. 2i, the ZnS:Cu-SG/PDMS film has a maximum tensile strain greater than 150% and a maximum load of approximately 6.2 kPa. Figure 2j shows the tensile stress–strain curves of the SFLC film under different maximum strains, the strains of the film increase linearly with the tensile forces. The closed hysteretic loops of the tensile stress–strain curves under different maximum strains demonstrate great recovery ability of the SFLC film, indicating reversible strain behavior during loading–unloading cycles. Figure 2k shows the cyclic tensile test of the SFLC film, which underwent 20 loading–unloading cycles under 50% strain (film width: 1 cm, stretching rate 50 mm min^−1^). After 20 cycles, the device did not show significant load degradation, indicating the mechanical stability of the SFLC sensor film. The excellent mechanical properties of the SFLC film make it suitable to serve as a flexible and wearable mechanoluminescent strain sensor for human (strain generated by normal human motion < 100%).

**Fig. 3:**
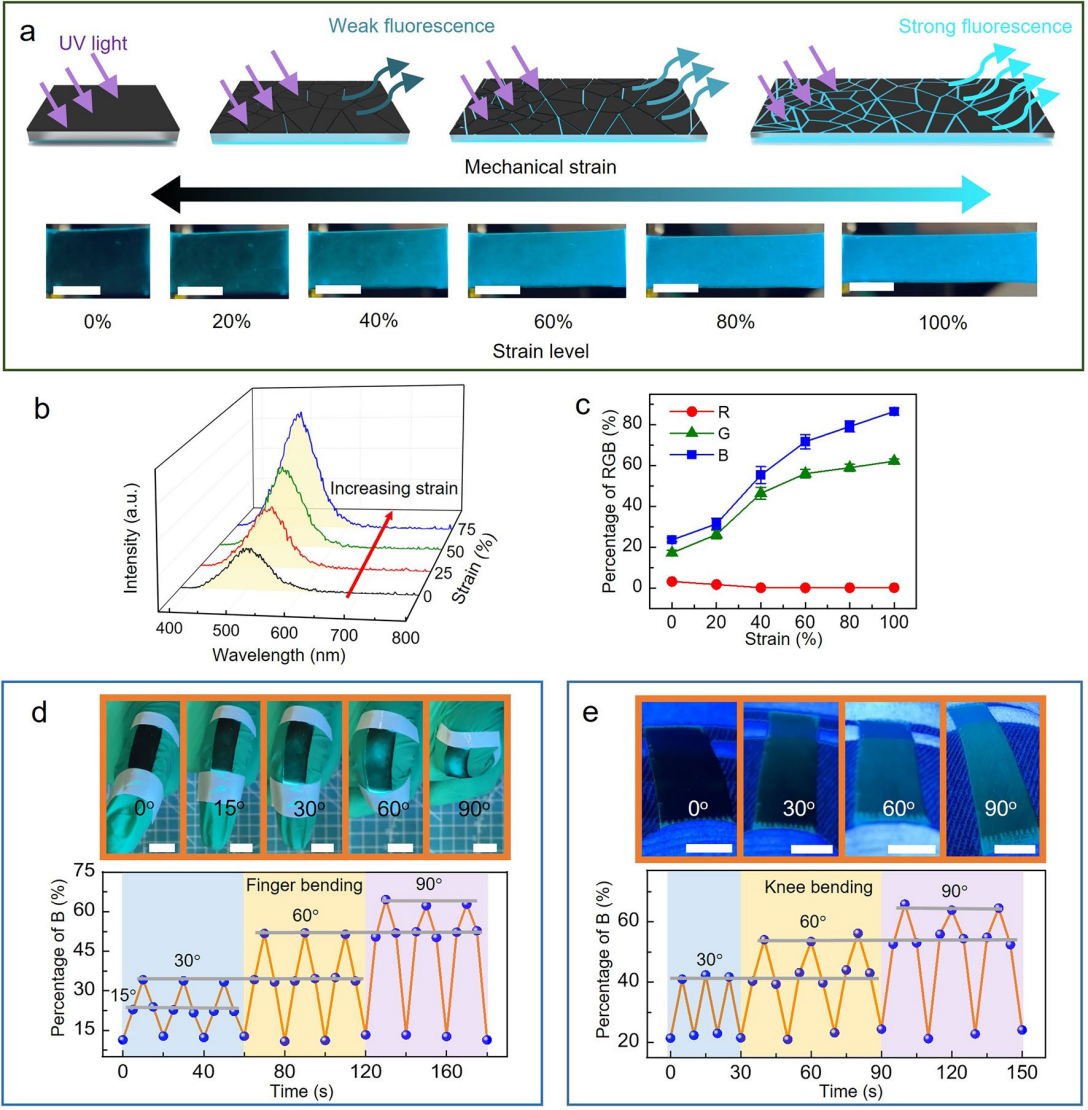
Schematic illustration showing the SFLC film as a strain sensor. **a** Sensing mechanism of the mechanoluminescent sensor, the intensity of fluorescence from the SFLC film increases with the increasing strain due to the increasing cracks of the CNTs layer on top, and photos of the SFLC film with different strain levels (scale bar: 1 cm). **b** Photoluminescence spectrum of the SFLC sensor film showing the increased intensity of photoluminescence with increasing strain. **c** Variation of the sensor RGB (%) with increasing strain (n = 5, mean ± s.d.). Photos showing **d** the bending of a finger and **e** the bending of knee with an attached SFLC strain sensor film and the resulting B (%) value under different finger bending and knee bending angles (scale bar: 1cm)

Figure 3a shows the working principle of the SFLC strain sensor. Under UV light irradiation, the fluorescent ZnS:Cu in the SG layer emits blue-green fluorescence. When the SFLC film is in relaxed state, the dense CNTs layer covered on top of the SFLC film shields the UV light and the emitted fluorescence from the bottom ZnS:Cu-SG layer. As a result, the detected fluorescence is very weak. When the SFLC film is under stretching, the fluorescence intensity of ZnS:Cu increases due to its intrinsic mechanoluminescent property. Moreover, the dense CNTs layer on top of the SFLC film cracks, generating slits on the top shielding layer, which allows UV light and the fluorescence from the bottom ZnS:Cu-SG layer to travel through the transparent PDMS middle layer and the cracks and slits on the top CNTs layer, leading to significantly increased intensity in the detectable fluorescence. With increasing strain level, the detectable intensities of fluorescence increase as more cracks and slits are generated on the top CNTs shielding layer. The photoluminescence (PL) spectrum (Fig. 3b) of the SFLC film under different uniaxial strain corroborates the theory mentioned above. With increasing strain, the intensity of the PL signal of the SFLC film increases. The change in the detectable fluorescence intensity of the SFLC film leads to different R, G, B color values extracted from the photos of the SFLC film captured by cameras. As shown in Fig. 3c, the percentage R, G, B values change with the increasing strain levels. By identifying the correlation between the detected percentage R, G, B values with the corresponding strains of the SFLC film, the strains of the film can be quantified by analyzing the R, G, B values of the color induced by strain from the photos of the SFLC film, which sets the foundation for the development of the AIFWMLS strain sensor system.

To optimize the strain sensing performance of the SFLC film, we studied the effects of number of lift-cast cycles of the top CNTs layers on the strain sensing performance of the SFLC film, since the thickness of the top CNTs layers increases with the number of the lift-cast cycles. Figure S6 shows that when the lift-cast cycle repeats 9 times, the sensitivity of the strain sensor is the highest, since the differences of R, G, B values obtained at 0% strain and 100% strain are the largest. We also studied the effects of ZnS:Cu concentration in SG layer on the performance of the SFLC film. As shown in Fig. S7a, the B (%) value is maximized when the ZnS:Cu concentration in SG layer is 10 wt%. The change of B (%) value is also the largest at 10 wt% ZnS:Cu concentration when the strain of the SFLC film is 100%, as shown in Fig. S7b. Therefore, 10 wt% ZnS:Cu concentration and 9 lift-cast cycles of the top CNTs layers are chosen to fabricate high-performance SFLC film. Moreover, we also studied the effects of PDMS layer deformation on the detected fluorescent intensity, since the change of PDMS layer thickness upon stretching could also affect the detectable fluorescence intensity. Results shown in Fig. S8 demonstrate that the stretching of the ZnS:Cu-SG/PDMS at strain range of 0–100% did not change the percentage R, G, B values, indicating that the deformation of PDMS will not affect the intensity of the detectable fluorescence in the SFLC film. To demonstrate the performance stability of the sensor, we studied the effects of repetitive stretching on the surface morphology and fluorescence intensity of the SFLC film under strain through SEM and PL spectroscopy. Interestingly, the initial gaps and slits between the CNTs clusters are reduced after 100 stretching cycles (Fig. S9a, b). The stretching of the film produces cracks on the top CNTs film, and some of the CNTs on the topmost of the original film could fill into these stretching-induced cracks due to the vibration of the deforming film, leading to a denser distribution of CNTs and less gaps and slits between CNTs clusters. Compared with the film after 100 stretching cycles, the surface morphology of the SFLC film after 300 and 500 stretching cycles did not change significantly since the stretching-induced cracks have been partially filled by CNTs during the initial 100 stretching cycles (Fig. S9c, d), which also demonstrates the stable adhesion of CNTs on the PDMS layer. The PL spectrum of the SFLC film under the same strain after different stretching cycles (Fig. S9e, f) corroborates with our proposed theory. The peak intensity of the PL spectrum slightly decreased after 100 stretching cycles, which could be due to the surface morphology change after the initial stretching. The denser CNTs layer produces less cracks under strain, leading to decreased detectable fluorescence intensity. After 100 stretching cycles, the detectable fluorescence intensity did not change significantly ( < 2.5%), since the surface morphology of CNTs layer as well as its shielding effect is stabilized.

A proof-of-concept study of using the SFLC film for recognition of finger bending and knee bending angles shows the great potential of the SFLC sensor film for human gesture recognition. As shown in Fig. 3d, when attaching the SFLC sensor film on the knuckles of the index finger, the B (%) values of the SFLC film increase with the increasing bending angles of the finger, since bending of the finger increases the strain of the SFLC film and thus the intensity of the detectable fluorescence. A strong correlation between the B (%) values and the finger bending angles can be observed **(**Fig. 3d). Similarly, B (%) values of the SFLC film attached on the knee increase with the increasing knee bending angles (Fig. 3e), and a strong correlation between the B (%) values and the knee bending angles can also be identified. The above study shows the great potential of the SFLC sensor film to serve as wearable sensors for human gesture recognition.

### Development of the Artificial Intelligence-Assisted Color Data Processing System

**Fig. 4:**
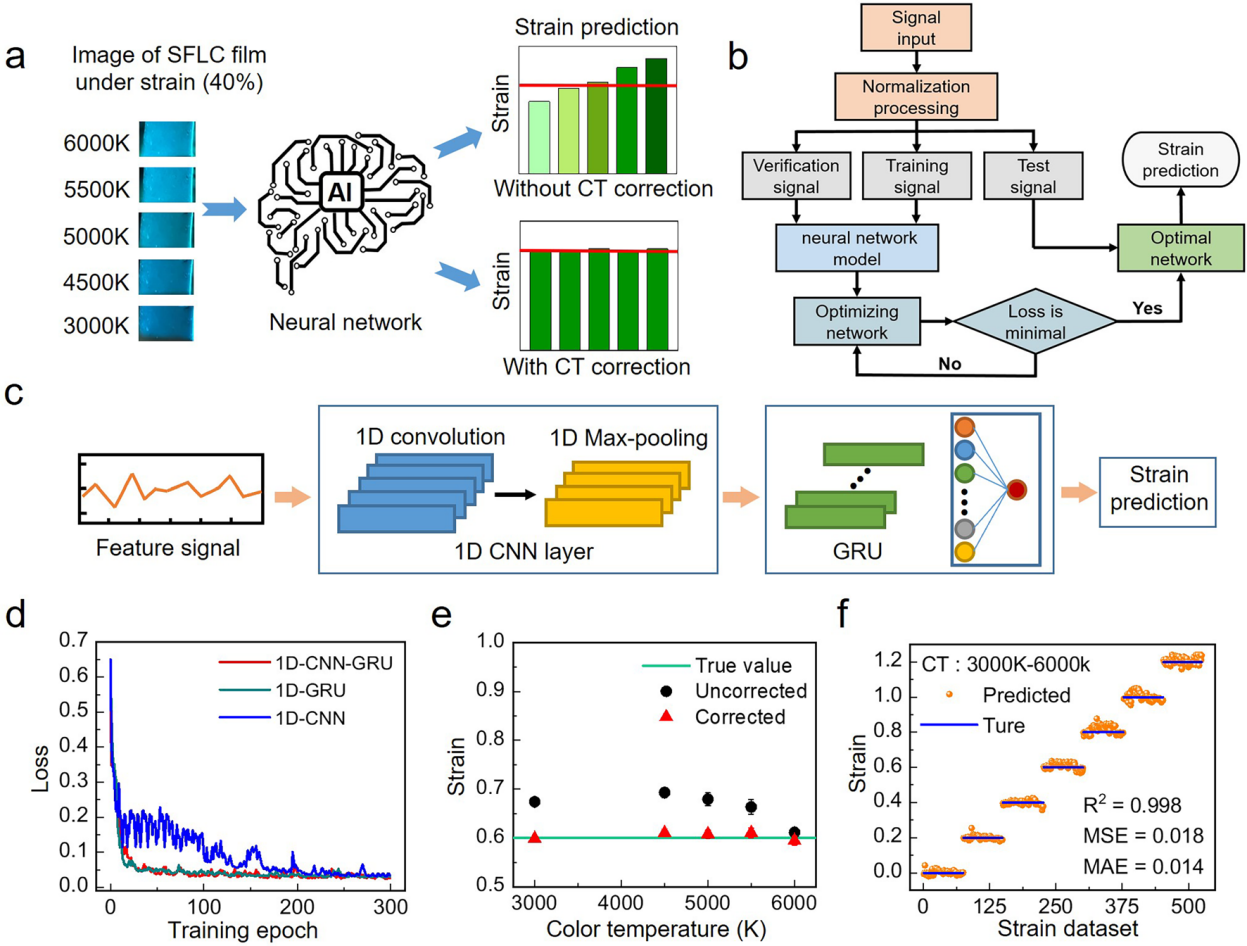
Development of the color data processing system. **a** Schematic illustration showing the concept of SFLC film strain sensing with the assistance of deep learning neural network-based artificial intelligence. After color temperature (CT) correction by the deep learning neural network, the accuracy of the predicted strain is significantly improved (red lines in the bar charts indicate the true strain value). **b** Algorithm flow chart showing the training and optimization process of the deep learning neural network for strain prediction with color temperature auto-correction. **c** Structure diagram of single channel CNN-GRU model with the feature signals extracted from the SFLC film as input and predicted strain values as output. **d** Loss value versus training epoch of the training process of the 1D-CNN-GRU, 1D CNN, and 1D GRU neural networks. **e** Uncorrected (black) and color temperature corrected strain data (red) compared with the true strain (green line) of the SFLC film (n = 3, mean ± s.d.). **f** Comparison of the predicted strains (yellow dots) obtained under different color temperature with the true strain values (blue line) with color temperature auto-correction by the CNN-GRU neural network

Despite the advantages of the MC/ML strain sensors such as wireless, battery-free, and easy fabrication, a major challenge that hindering the practical application of wearable MC/ML strain sensors is the fast and accurate interpretation of color data to strain values. Moreover, the ambient light condition could be different for different measurements, as shown in Fig. 4a. The changing color temperature could lead to significant discrepancies between the interpreted strains and the true strain values. To tackle these challenges, this work develops a SFLC CDPS based on CNN-GRU deep learning neural network for the fast and accurate interpretation of color data acquired from SFLC sensor with color temperature auto-correction. To develop the CDPS system, firstly, a smart phone camera captures image of SFLC sensor under different color temperatures (3000, 4500, 5000, 5500, and 6000 K). Then, Open Library for Images (OpenCV) extracts the features from the region of interest (ROI) in the image (data feature listed in Table S1), forming the dataset for the neural network training. To establish the correlation between the sensor strain and the resulting strain-induced colors for strain prediction, we train the CNN-GRU neural network using the collected color data under different strains and color temperatures. We randomly divided the dataset into a training set, a validation set, and a test set in a ratio of 3:1:1 (Fig. 4b). Figure 4c shows the basic structure of the single channel CNN-GRU (1D-CNN-GRU) neural network, which is composed of one-channel CNN, two layers of GRUs, and a fully connected layer (Dense), with the feature signal extracted from the SFLC film as input and predicted strains as output (neural network parameters listed in Table S2). The linear function in the fully connected layer enables the regression prediction of strain. Using mean absolute error (MAE) as hyperparameters for model optimization, after 300 iterations, the loss value curves converge for the neural network models studied in this work, including 1D-CNN-GRU, 1D-CNN, and 1D-GRU (Fig. 4d), which completes the training process for the neural network model. The loss value of 1D-CNN-GRU is the smallest among the three models. Integration of CNN and GRU combines the advantages of both neural network of CNN and GRU, which improves the capability of the hybrid neural network to understand the sequence data by modeling the spatiotemporal relationship in the sequence data more comprehensively. Compared to other complex neural networks, the hybrid CNN-GRU neural network is easy to train and use, thanks to its simple structure with fewer parameters. The coefficients of determination (R^2^) for strain prediction of the 1D-CNN-GRU neural network (0.998) is higher than those of the 1D-CNN and 1D-GRU neural networks (Fig. S10a), indicating the better accuracy of the 1D-CNN-GRU in strain prediction of the SFLC film than 1D-CNN and 1D-GRU neural networks. To achieve auto-correction of the errors in the predicted strain data caused by the varying color temperature in different measurements, we train the CNN-GRU neural network with color data obtained under different color temperatures. Figure 4d shows the comparison of the predicted strain data with (red) and without color temperature correction at a fixed strain level (60%). The differences between the predicted strain values with color temperature correction (red dots) and the true strain value (green line) of the SFLC film are much smaller than those between the uncorrected ones (black dots) and true strain value. Comparison of the predicted strain values with (Fig. 4e) and without (Fig. S10b) color temperature correction with the true strain values at different strain levels further demonstrates that the CDPS system significantly improves the accuracy of the predicted strains. After color temperature correction by the CDPS system, the R^2^ value increased from 0.929 to 0.998, and the MSE (mean square error) and MAE (mean absolute error) values decreased down to 0.018 and 0.014, respectively. Results shown in Fig. 4 demonstrate that the CDPS system based on CNN-GRU neural network is a powerful tool for the fast and accurate interpretation of color data into strain values for the wearable SFLC strain sensor. The color of the SFLC film changed instantaneously upon stretching without any delay. And it took about 15 s (Movie S1, photo uploaded to the system at 0:00:16, strain results displayed at 0:00:31) for the AIFWMLS system to generate the strain prediction result and display it on the smart phone. Moreover, we studied the effects of different models of mobile phones on the performance of the sensor system for strain detection. We used smart phones of three different brands (Apple, Xiaomi, Vivo) to capture the image of a SFLC film under different strains (0, 20%, 40%, 60%, 80%, 100%). For each strain, we take 10 photos using each brand of smart phone. Then, we use the CDPS system to predict the strain values of the SFLC film using photos taken by smart phones of different brands. The results are shown in Table S3 and Fig. S11. Figure S11a shows that the RGB values extracted from the photos of the SFLC film taken by smartphones of different brands (Apple, Xiaomi, Vivo) only show negligible differences. The small RMSE and MAE values and large R^2^ values (all above 0.99) for strain prediction by the neural network using photos taken by different brands of smart phones (Fig. S11b) indicate that the model of smart phones used for color data collection has very little influence on the performance of the AIFWMLS sensor system.

To further prove the insensitivity of the performance of the AIFWMLS sensor system to bending and twisting, we took pictures of the SFLC film after different bending and twisting cycles and use the CNN-GRU neural network to predict strains from these photos. The performance of the sensor system after different bending and twisting cycles is shown in Tables S4 and S5. After repetitive bending and twisting, the RSME, MAE, and R^2^ value for strain prediction from the SFLC film only show very small changes, and the R^2^ values are all above 0.99 which indicates high strain prediction accuracy, proving that the sensor system is insensitive to bending and twisting. To demonstrate the repeatability of the as-proposed sensor for strain deformation, we test a SFLC film repetitively after multiple times of bending, twisting, and stretching. The resulting strain values predicted by the CNN-GRU neural network from these repetitive tests are shown in Fig. S12. All the strain values obtained from these repetitive tests did not significantly deviate from the true strain value (0.6) and the variations of the predicted strain values after hundred times of bending, twisting, and stretching are very small, demonstrating the excellent repeatability of the as-proposed sensor.

### Applications of the AIFWMLS System

**Fig. 5:**
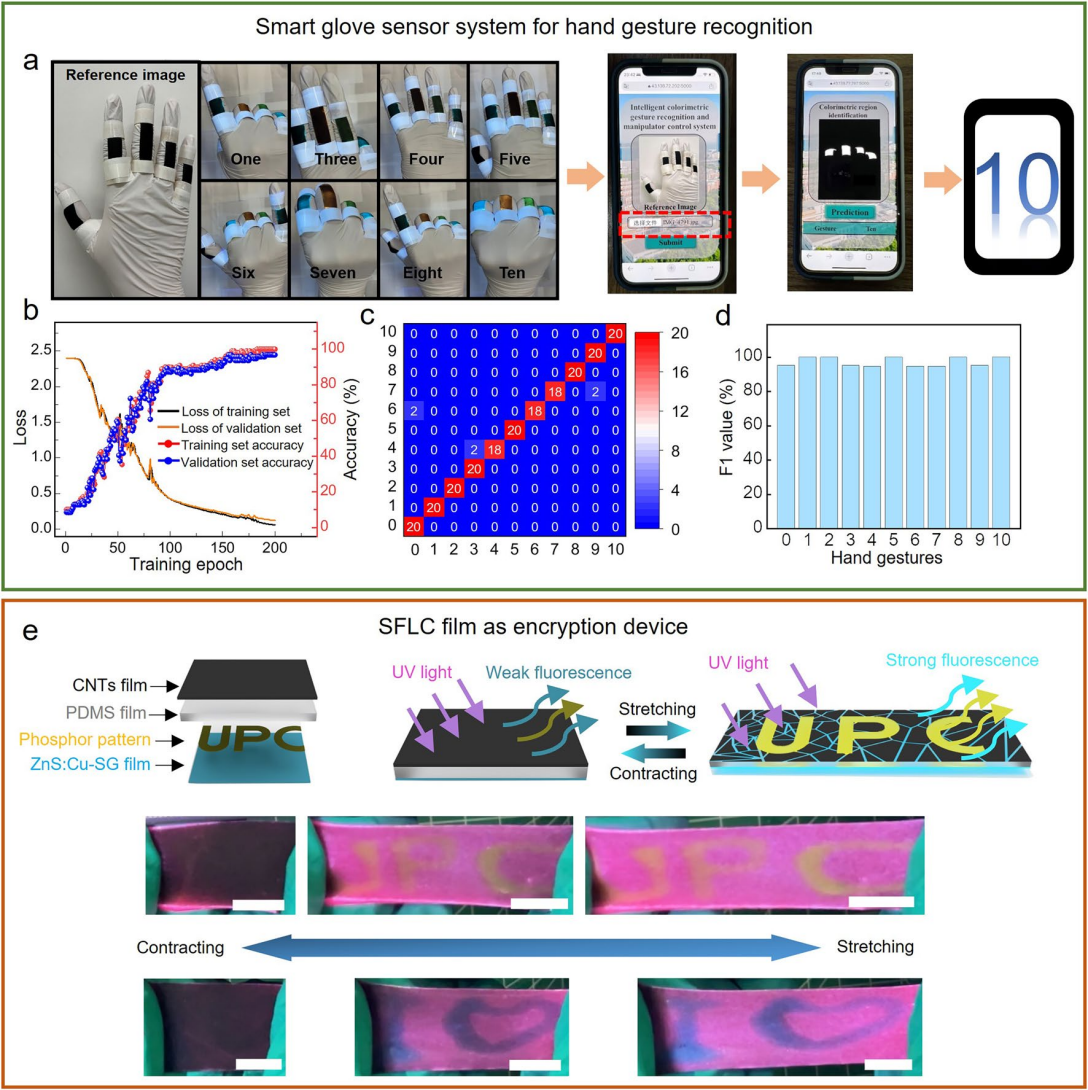
Applications of the SFLC film. **a** The flow chart showing the process of hand gesture recognition using the smart glove. **b** The variation of loss value and accuracy during the training process of the neural network. **c** Confusion map of the classification of the 11 hand gestures. **d** Evaluation index of the hand gesture classification model. **e** Schematic illustration of the SFLC film as encryption device including the structure of the SFLC encryption film, the working principle of the SFLC encryption film, and photograph showing the concealed “UPC” and “I ♥” logo emerged upon stretching the SFLC encryption film in the horizontal direction

To demonstrate the application potential of the AIFWMLS sensor system, we developed a smart glove wearable strain sensor array based on the sensing scheme of the AIFWMLS system for hand gesture recognition. The smart glove wearable sensor array is composed of five pieces of SFLC sensor film attached on the finger knuckle positions on the glove using 3 M medical adhesives (Fig. 5a). The fluorescent layer in the SFLC films attached on the knuckles of the index finger and the little finger is ZnS:Cu-SG, which is the same with the SFLC film showed in Figs. 2 and 3. To better distinguish different hand gestures, for the SFLC films attached on the knuckles of the middle finger and the ring finger, we substitute the ZnS:Cu fluorescent materials in the SG layer of SFLC films with commercial orange and green fluorescent powders, respectively. The smart glove wearable strain sensor system can be integrated with the cloud-server-based data collection and processing system and the web browser user interface for data acquisition, processing and results display, which was developed in our previous work [47]. A smart phone captures the image of the hand wearing the smart glove sensor array through its camera. The varying bending angles of the finger knuckles of different hand gestures lead to different color combinations in the SFLC films on the smart glove. A web browser user interface uploads the captured image of the smart glove to a cloud-server, where the image is processed and analyzed by a trained multichannel CNN-GRU deep learning neural network. The system predicts the hand gesture based on the extracted color data from the image of the smart glove and then returns the predicted hand gesture back to the web browser for results display. Figure S13 shows the training process of the CNN-GRU neural network used for hand gesture prediction. The data processing system extracts the colors of the five SFLC films on the smart glove from the images of a human hand wearing the smart glove posing different hand gestures (Chinese hand number gestures 0–10, representative gestures shown in Fig. 5a). To construct the dataset for training of the CNN-GRU neural network for hand gesture recognition, the data processing system extracts the colors of the five SFLC films on the smart glove and converts the color data to a RGB frequency distribution, which shows the image intensity distribution over RGB values (an example is shown in Fig. S14). There are five SFLC films on each finger of the smart glove sensor, providing five groups of RGB data for the training of the 5D-CNN-GRU neural network for hand gesture recognition. As demonstrated in Fig. 4, the varying color temperature affects the obtained RGB value from the captured image of the SFLC film, which could increase the chances of misinterpretation in the hand gestures. To tackle the negative effects caused by the varying color temperature in hand gesture recognition, we collect 11 groups of data from the smart glove for Chinese number gesture 0–10 under different color temperatures (3000, 4500, 5000, 5500, and 6000 K) for training of the deep learning neural network. The feature data from each of the five SFLC films on each finger serve as the input signal for each channel in the 5D-CNN-GRU neural network, which is randomly divided into a training set, a validation set and a test set for model training and optimization in a ratio of 3:1:1. The number of neurons for classification in the Softmax function of the fully connected layer is 11, which is the same with the number of hand gestures to recognize, enabling the recognition of the 11 Chinese number gestures. The training process completes after 200 iterations until the loss value (cross-entropy loss) curves converge (loss values are the smallest) and the accuracy reaches 98%, as shown in Fig. 5b (parameters of the 5D-CNN-GRU neural network shown in Tables S6 and S7). The profusion matrix obtained using the test dataset in Fig. 5c shows the performance of the trained 5D-CNN-GRU neural network with overall accuracy of 97.3% for recognition of the 11 Chinese number hand gestures. F1 value is an important parameter to evaluate the performance the 5D-CNN-GRU neural network for classification. Figure 5d shows the F1 values for the recognition of the 11 hand gestures, which are all in a range from 94.5%-100%, demonstrating the excellent performance of the 5D-CNN-GRU neural network for hand gesture recognition. Movie S2 shows the entire process and the setup of hand gesture recognition using the AIFWMLS system. It took about 10 s (photo of smart glove uploaded to the system at 0:00:17, recognition results displayed at 0:00:27) for the system to generate the strain prediction result and display it on the smart phone, demonstrating the fast response of the AIFWMLS system for hand gesture recognition.

Another potential application of the SFLC film is encryption devices (Fig. 5e). As a proof-of-concept, we encoded information “UPC” and “I ♥” in the SFLC film by writing the above letters and characters (“UPC” and “I ♥”) on the bottom fluorescent SG layer using inks made by mixing SG and commercial fluorescent powders of different colors (yellow powder for “UPC” and blue powder for “I ♥”). When the SFLC film is in relaxed state, the dense top CNTs layer shields the yellow and blue fluorescence from the encoded letters and characters at the bottom. As a result, the encoded information cannot be observed. A mechanical cue, which is the stretching of the SFLC encryption film, uncovers the encoded information under UV light. Upon stretching, the transmittance of the yellow and blue fluorescence from the encoded letters and characters significantly increases as the top CNTs layer cracks and slits grow. As a result, the SFLC film displays the encoded letters and characters upon stretching under UV light. Therefore, the SFLC film can serve as a potential encryption device, and stretching of the device under UV light is the cue to reveal the concealed information.

## Conclusions

In summary, this work demonstrates the powerful integration of deep learning neural network with flexible mechanoluminescent film as a wearable, wireless, battery-free sensor system (AIFWMLS) for rapid and accurate detection of strains. The sandwich-structured flexible mechanoluminescent film (SFLC) developed in this study shows remarkable and robust mechanoluminescent performance with a simple sensor structure, which is easy to fabricate. The development of color data processing system (CDPS) based on the deep learning neural network artificial intelligence can rapidly and accurately extract and interpret the color data from the SFLC film to strain values with auto-correction of errors caused by the varying color temperature in different measurements. The proof-of-concept smart glove mechanoluminescent sensor system based on the AIFWMLS system demonstrates the huge potential of the artificial intelligence-assisted MC/ML sensor in human gesture recognition. Moreover, a demonstration of the SFLC film as a potential encryption device further shows its multifunctionality. The integration of deep learning neural network-based artificial intelligence and flexible mechanoluminescent film provides a promising strategy to break the “color to strain value” bottleneck that hinders the practical application of flexible MC/ML strain sensors, which could promote the development of wearable and flexible MC/ML strain sensors from laboratory research to consumer markets.

## Supplementary Information

Below is the link to the electronic supplementary material.

Supplementary file1 (DOCX 3775 KB)

Supplementary file2 (MP4 49897 KB)

Supplementary file3 (MP4 41550 KB)

Supplementary file4 (MP4 55061 KB)
