# Supplementary material for: An Artificial Intelligence-Assisted Flexible and Wearable Mechanoluminescent Strain Sensor System
Source: Nanomicro Lett. 2024 Nov 15;17:62. doi: 10.1007/s40820-024-01572-5 (PMC11564496; doi:10.1007/s40820-024-01572-5)
Supplement: Supplementary file 1 — Supplementary file1 (DOCX 3775 KB) [file 40820_2024_1572_MOESM1_ESM.docx]

Supporting Information for

**An Artificial Intelligence-Assisted Flexible and Wearable Mechanoluminescent Strain Sensor System**

Yan Dong^1,^ *, Wenzheng An^1^, Zihu Wang^1^ and Dongzhi Zhang^1,^*

^1^College of Control Science and Engineering, China University of Petroleum (East China), Qingdao 266580, P. R. China

*Corresponding authors. E-mail: [dzzhang@upc.edu.cn](mailto:dzzhang@upc.edu.cn) (Dongzhi Zhang); [yandong@upc.edu.cn](mailto:yandong@upc.edu.cn) (Yan Dong)

**Supplementary Figures**


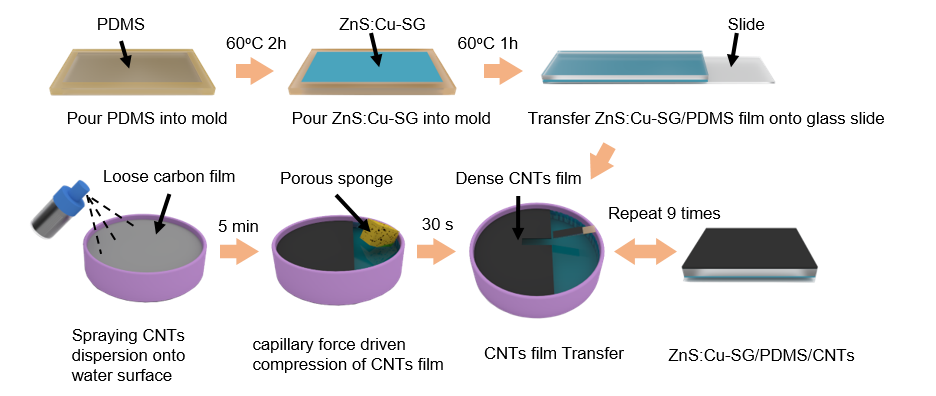


**Fig. S1** Schematic illustration of the preparation process of SFLC film


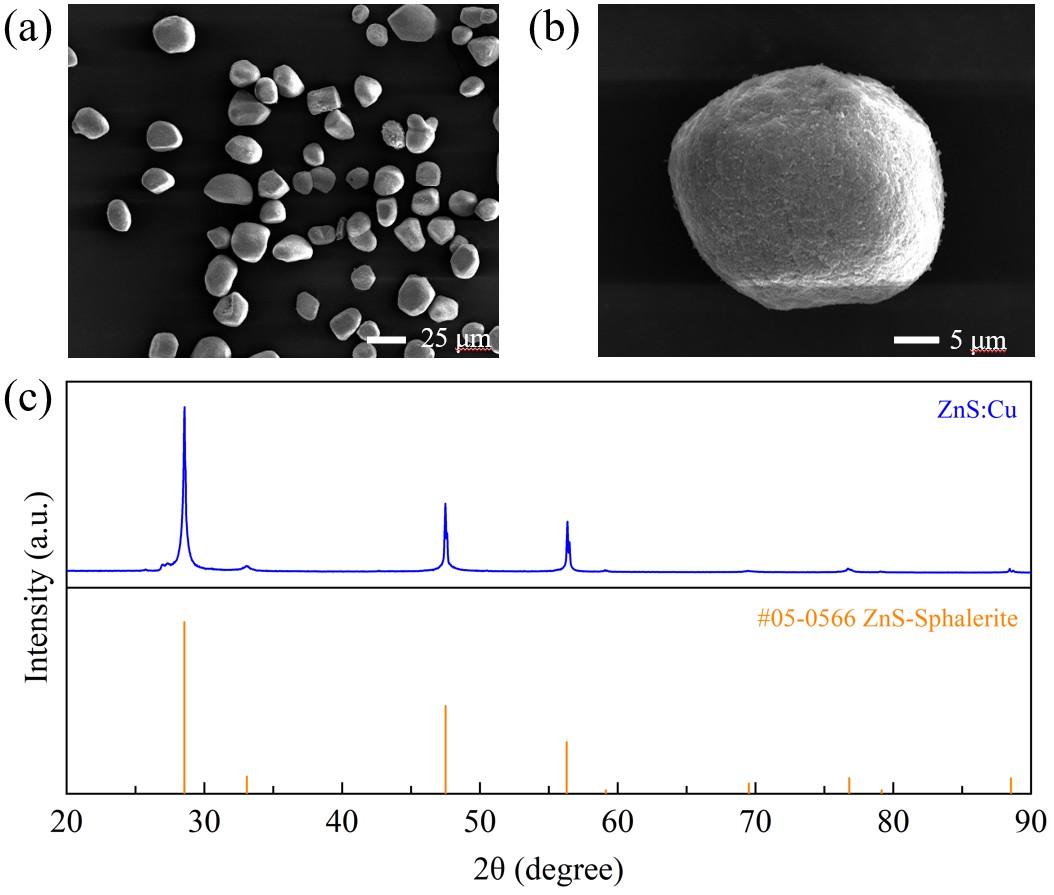


**Fig. S2** (**a**) and (**b**) SEM image of the ZnS:Cu fluorescent particles for the fabrication of the SFLC film. (**c**) XRD of the ZnS:Cu fluorescent particles


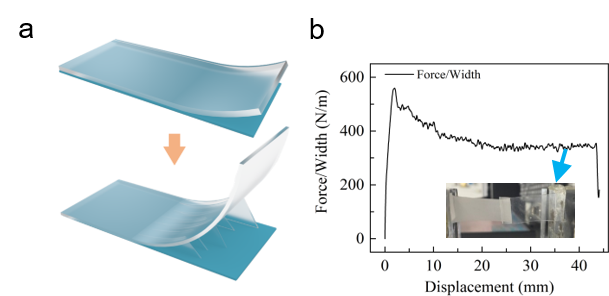


**Fig. S3** (**a**) Schematic illustration of the ZnS:Cu-SG/PDMS film stripping test. (**b**) Stripping force vs displacement curve of the ZnS:Cu-SG/PDMS film


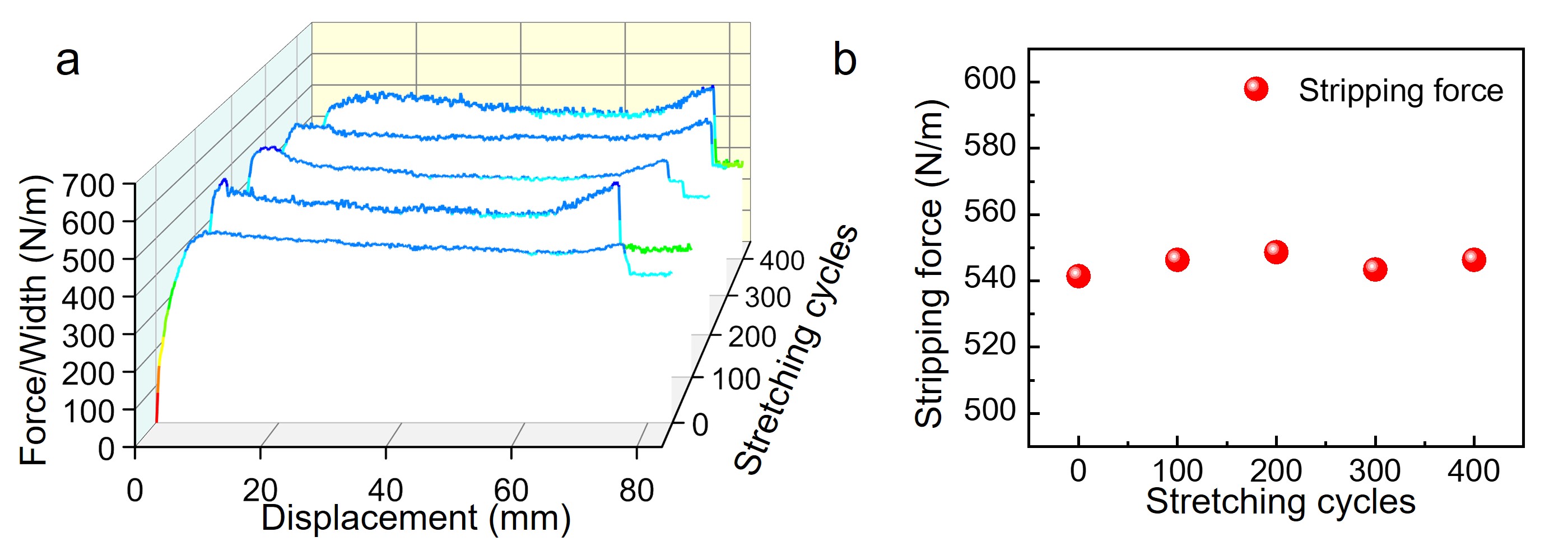


**Fig. S4** (**a**) Stripping force *vs* displacement curves of the SFLC film after different stretching cycles. (**b**) Stripping force at the beginning of detachment of PDMS layer and the SG layer of the SFLC film


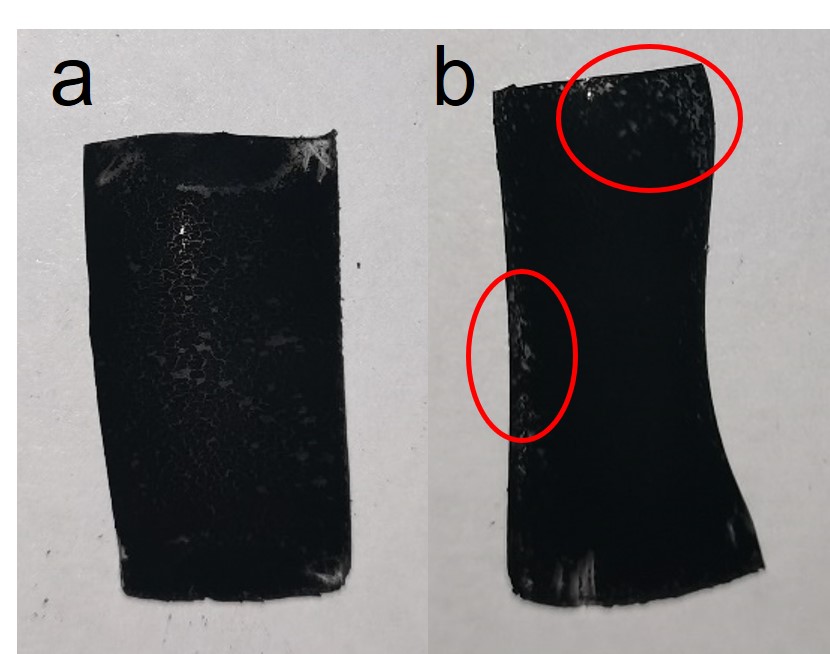


**Fig. S5** Photos of SFLC film fabricated by different methods (**a**) drop-casting method (**b**) brush-casting method, the CNTs film in the red circles is not uniform


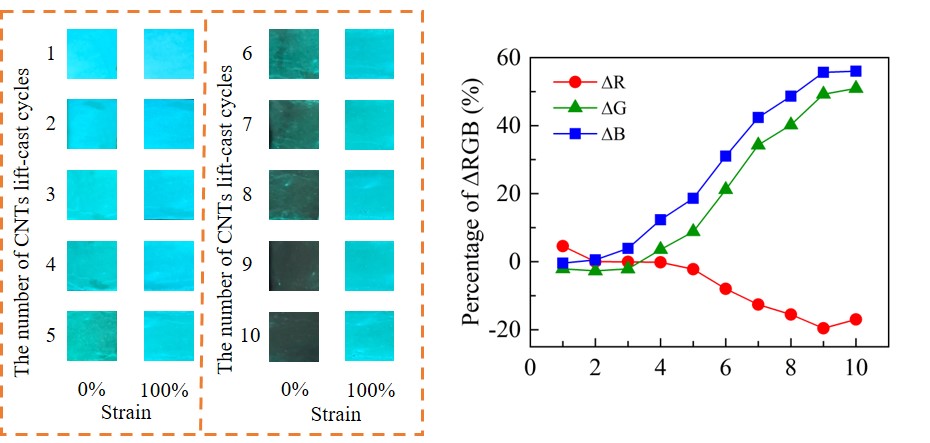


**Fig. S6** Effects of number of lift-cast cycles of the top CNTs layers on the strain sensing performance of the SFLC film


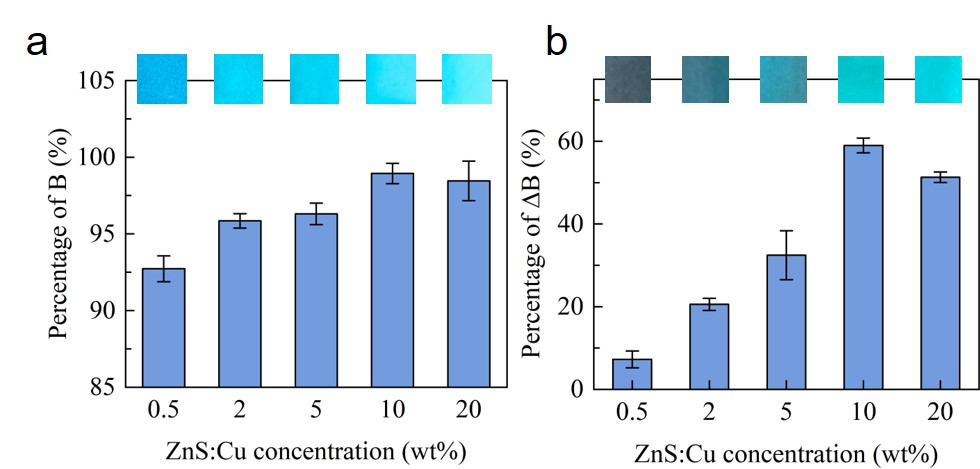


**Fig. S7** Effects of ZnS:Cu concentration in the SG layer on the performance of the SFLC film. (n = 5, mean ± s.d.)\


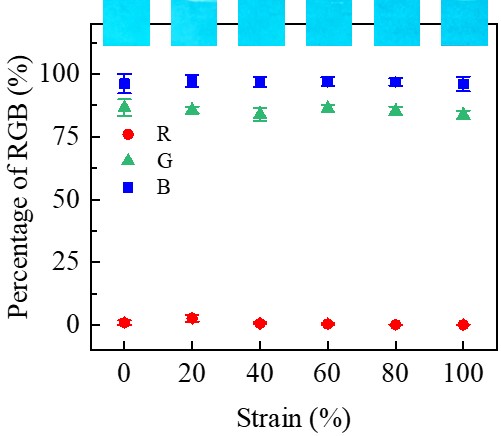


**Fig. S8** Effects of PDMS layer deformation on the detected fluorescent intensity


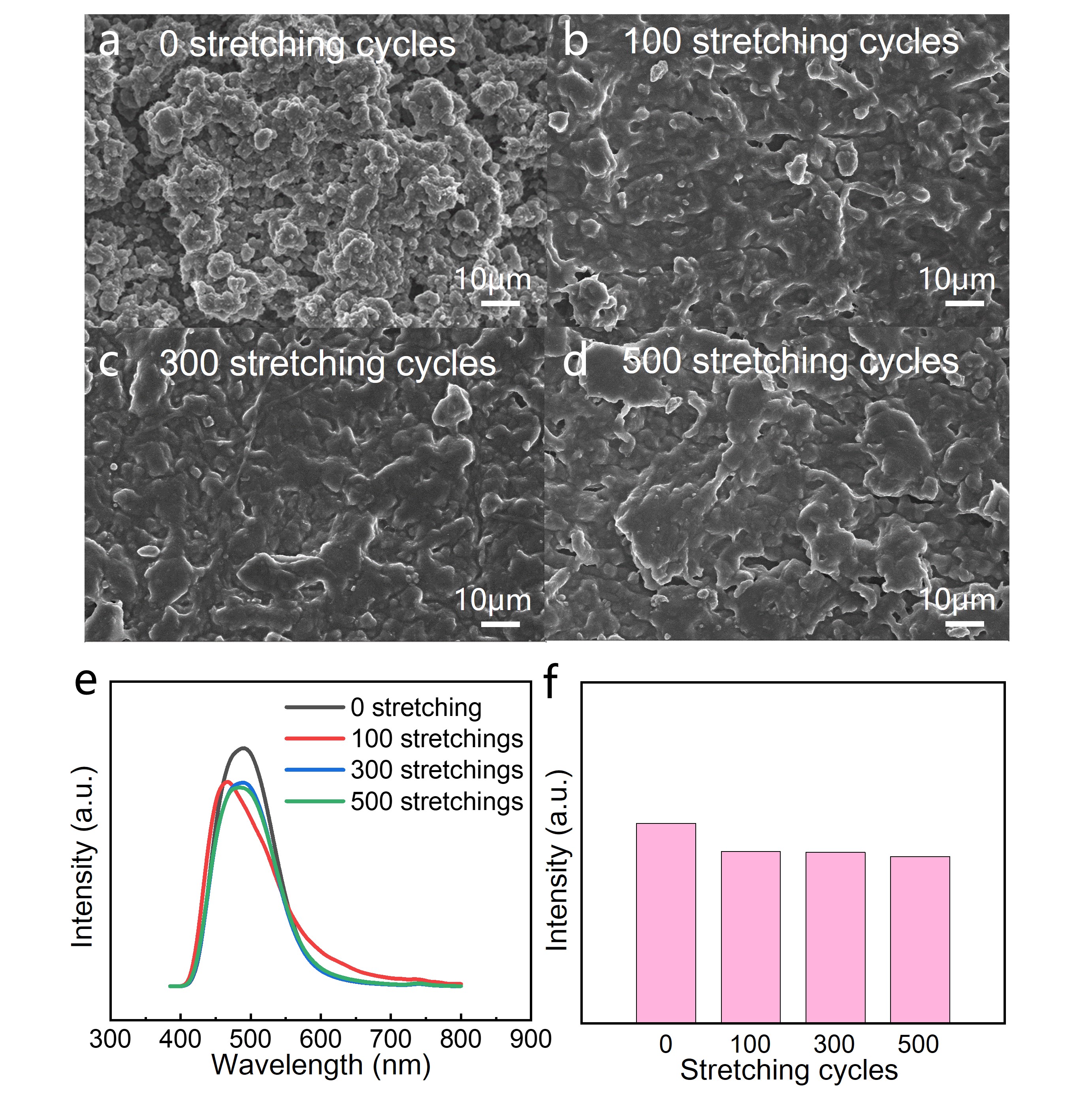


**Fig. S9** SEM images showing the surface morphology of the top CNTs shielding layer after (**a**) 0, (**b**) 100, (**c**) 300, and (**d**) 500 stretching cycles. (**e**) PL spectrum and (**f**) comparison of the peak intensity of the PL spectrum of the SFLC film under the same strain after different stretching cycles

**Table S1** List of features for the color data for neural network training and strain prediction

| No. | Feature | Description | No. | Feature | Description |
| --- | --- | --- | --- | --- | --- |
| 1 | R_m_ | Mean value of R channel | 13 | V_m_ | Mean value of V channel |
| 2 | G_m_ | Mean value of G channel | 14 | X_m_ | Mean value of X channel |
| 3 | B_m_ | Mean value of B channel | 15 | Y_m_ | Mean value of Y channel |
| 4 | R_m_/RGB_n_ | Ratio of R_m_ and RGB_n_ | 16 | Z_m_ | Mean value of Z channel |
| 5 | G_m_/RGB_n_ | Ratio of G_m_ and RGB_n_ | 17 | L_m_ | Mean value of L channel |
| 6 | B_m_/RGB_n_ | Ratio of B_m_ and RGB_n_ | 18 | a_m_ | Mean value of a channel |
| 7 | RGB_n_ | Sum of R_m_, G_m_, B_m_ | 19 | b_m_ | Mean value of b channel |
| 8 | Intensity  (unweighted) | Grey scale formula result  0.299*R_m_+0.587* G_m_+0.114* B_m_ | 20 | HSV_n_ | Sum of H_m_, S_m_, V_m_ |
| 9 | Cb_m_ | Mean value of C_b_ channel  -0.172*R_m_-0.339* G_m_+0.511* B_m_+128 | 21 | XYZ_n_ | Sum of X_m_, Y_m_, Z_m_ |
| 10 | Cr_m_ | Mean value of C_r_ channel  0.511*R_m_-0.428* G_m_-0.083* B_m_+128 | 22 | Lab | Sum of L_m_, a_m_, b_m_ |
| 11 | H_m_ | Mean value of H channel | 23 | Strain | Strain of the sensor |
| 12 | S_m_ | Mean value of S channel | 24 | CT | Color temperature |

**Table S2** Parameters of 1D-CNN-GRU model for strain prediction

| Layer | Name | Input  shape | Number of neurons | Convolution kernel | Step size | Return sequences | Activation function | Output shape |
| --- | --- | --- | --- | --- | --- | --- | --- | --- |
| 1 | InputLayer | 24×1 | － | － | － | － | － | 24×1 |
| 2 | Conv1D | 24×1 | 32 | (1,1) | 1 | － | Relu | 24×32 |
| 3 | MaxPooling1D | 24×32 | － | － | 1 | － | － | 24×32 |
| 4 | Conv1D | 24×32 | 64 | (1,1) | 1 | － | Relu | 24×64 |
| 5 | Conv1D | 24×32 | 64 | (1,1) | 1 | － | Relu | 24×64 |
| 6 | MaxPooling1D | 24×64 | － | － | 1 | － | － | 24×64 |
| 7 | Conv1D | 24×64 | 128 | (1,1) | 1 | － | Relu | 24×128 |
| 8 | Conv1D | 24×64 | 128 | (1,1) | 1 | － | Relu | 24×128 |
| 9 | MaxPooling1D | 24×128 | － | － | 1 | － | － | 24×128 |
| 10 | GRU | 24×128 | 64 | (1,1) | － | True | Linear | 24×64 |
| 11 | GRU | 24×64 | 64 | (1,1) | － | False | Linear | 64 |
| 12 | Dense | 64 | 1 | － | － | － | Linear | 1 |


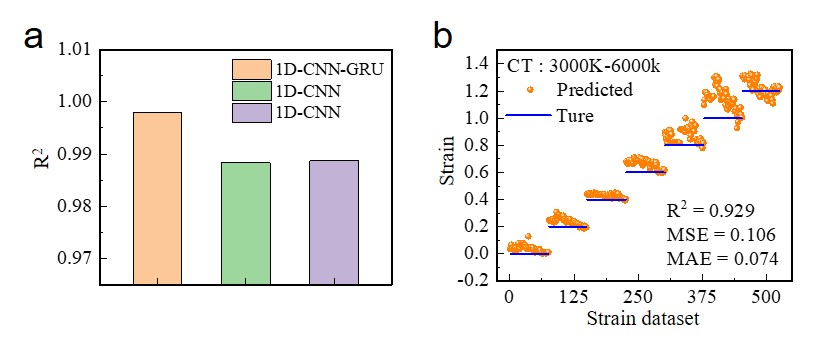


**Fig. S10** (**a**) Comparison of the coefﬁcient of determination (R^2^) of the output results of the three neural network models. (**b**) Comparison of the predicted strains (yellow dots) obtained under different color temperature with the true strain values (blue line) without color temperature auto-correction


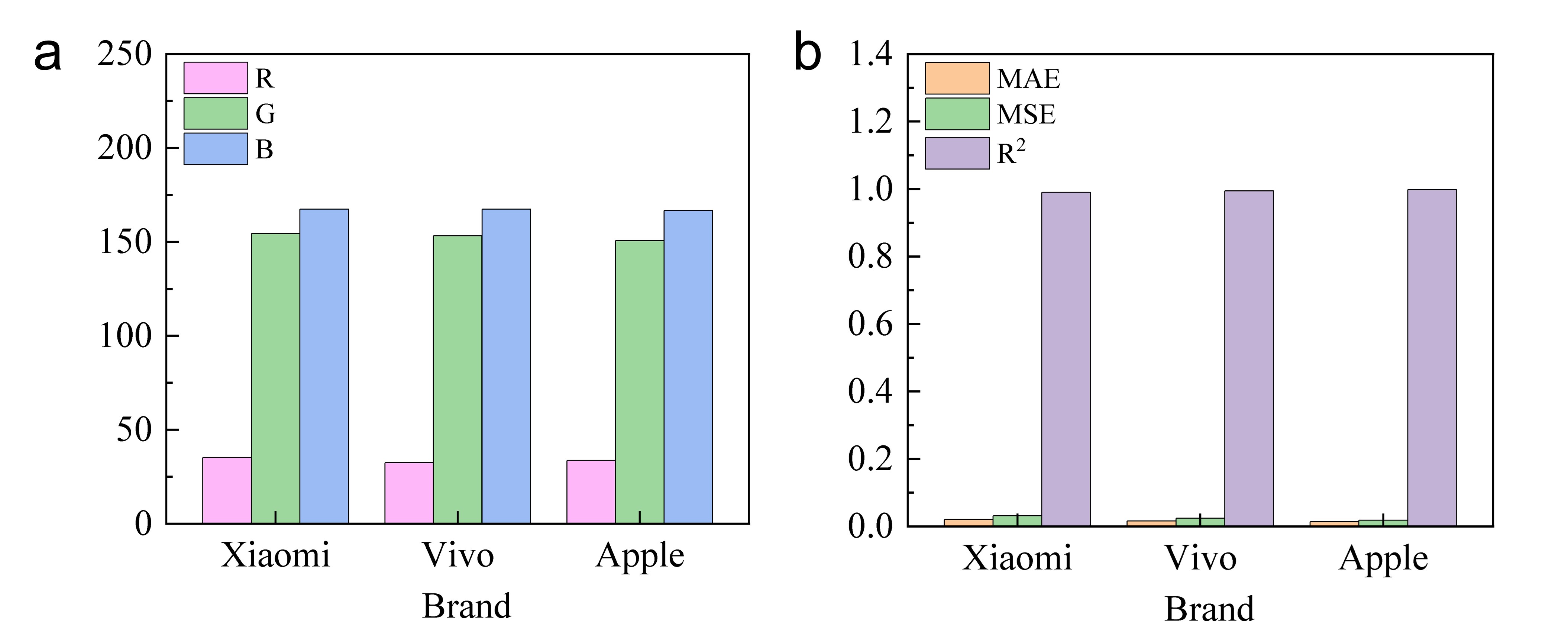


**Fig. S11** (**a**) RGB values extracted from phots of the same SFLC film taken by smartphones of different brands. (**b**) RMSE, MAE, and R^2^ values obtained using photos taken by smartphones of different brands

**Table S3** RMSE, MAE, and R2 values obtained using photos taken by smartphones of different brands

**
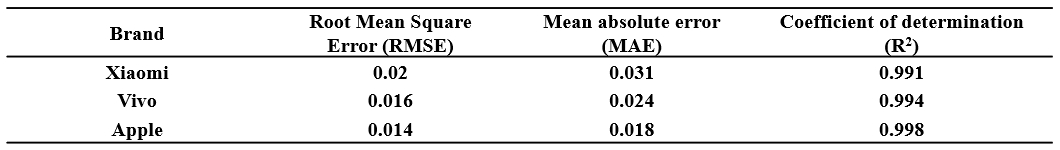
**

**Table S4** Effects of repetitive bending on the strain prediction performance of the sensor system


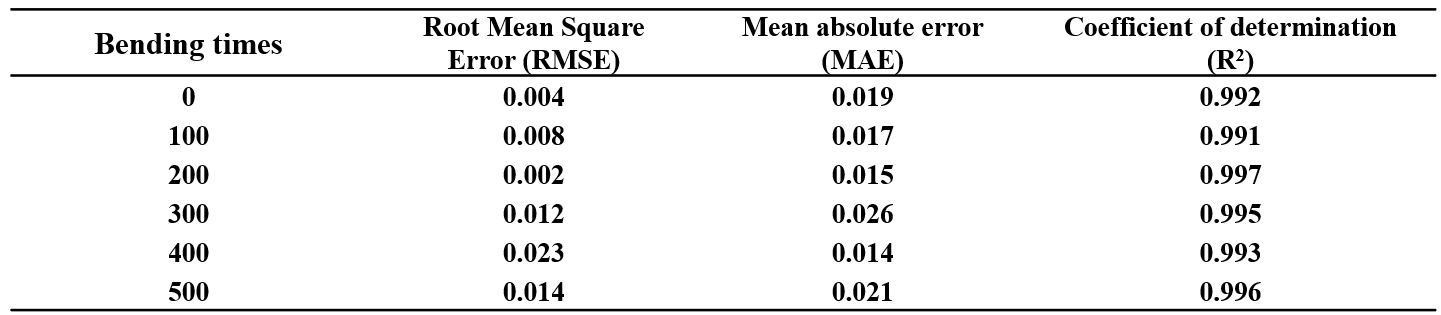


**Table S5** Effects of repetitive twisting on the strain prediction performance of the sensor system


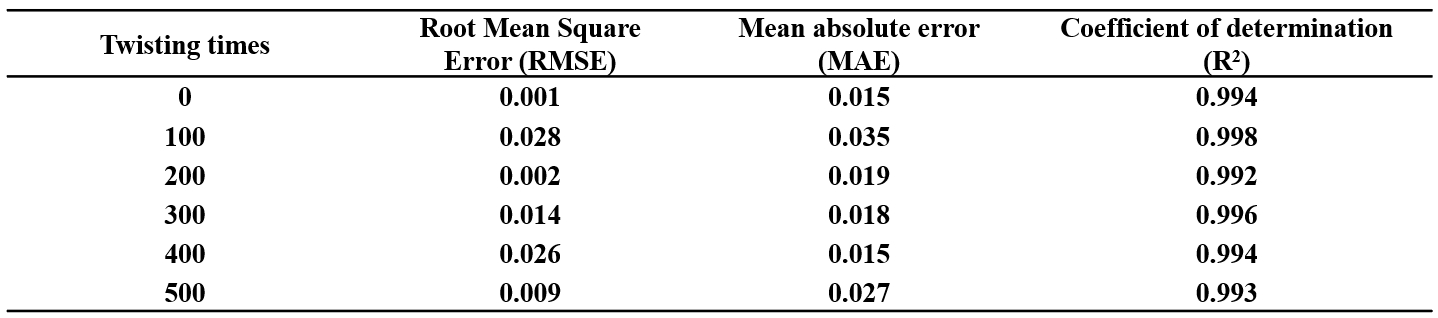


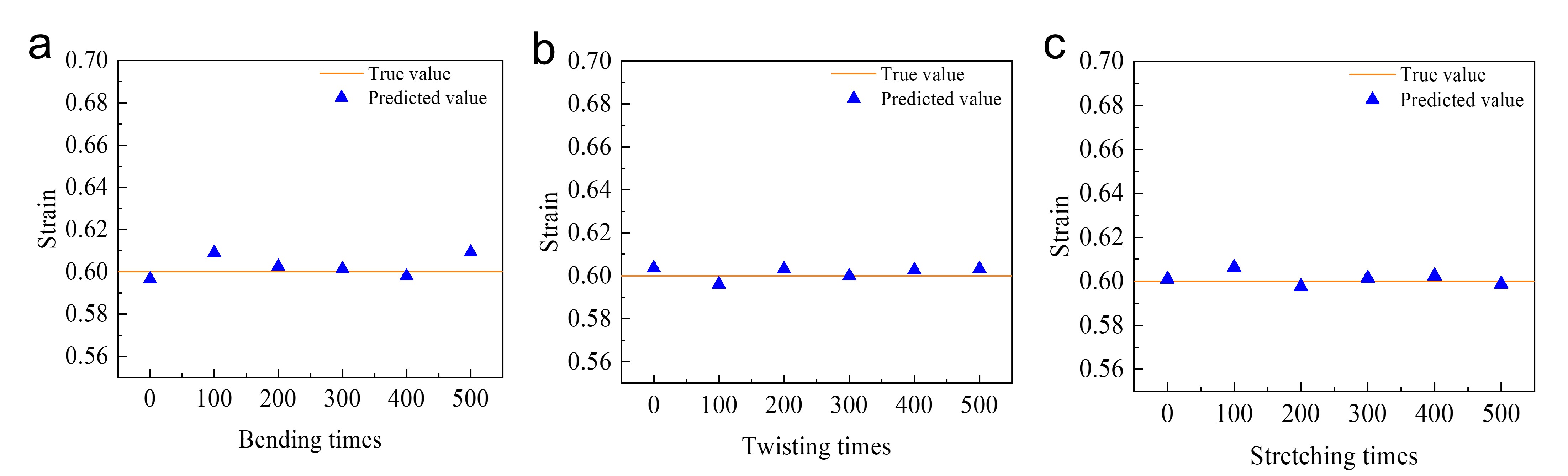


**Fig. S12** The strain prediction results of repetitive testing of the sensor film after multiple times of (**a**) bending, (**b**) twisting, and (**c**) stretching


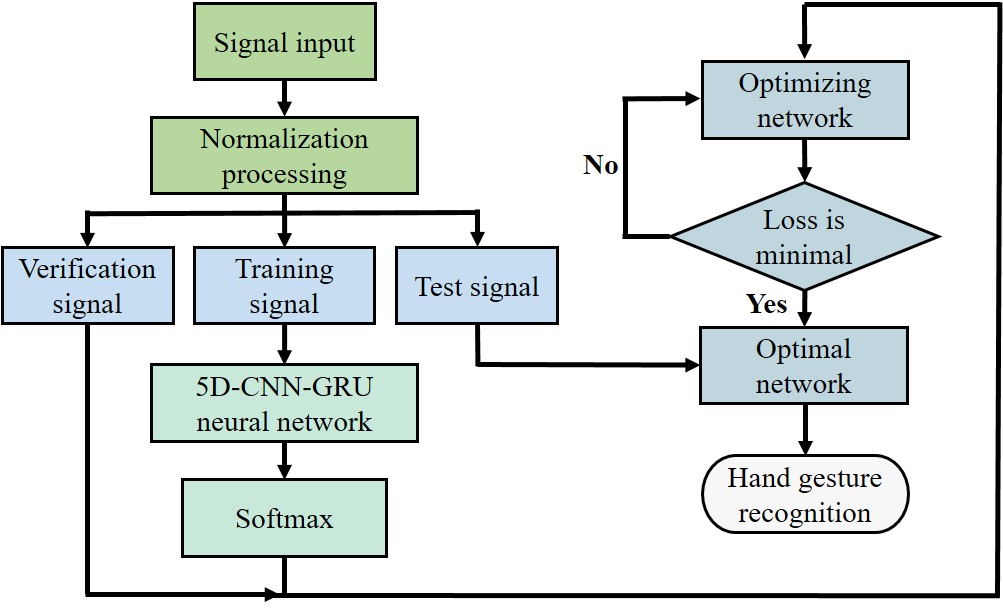


**Fig. S13** Algorithm flow chart showing the training and optimization process of the 5D-CNN-GRU neural network for hand gesture recognition


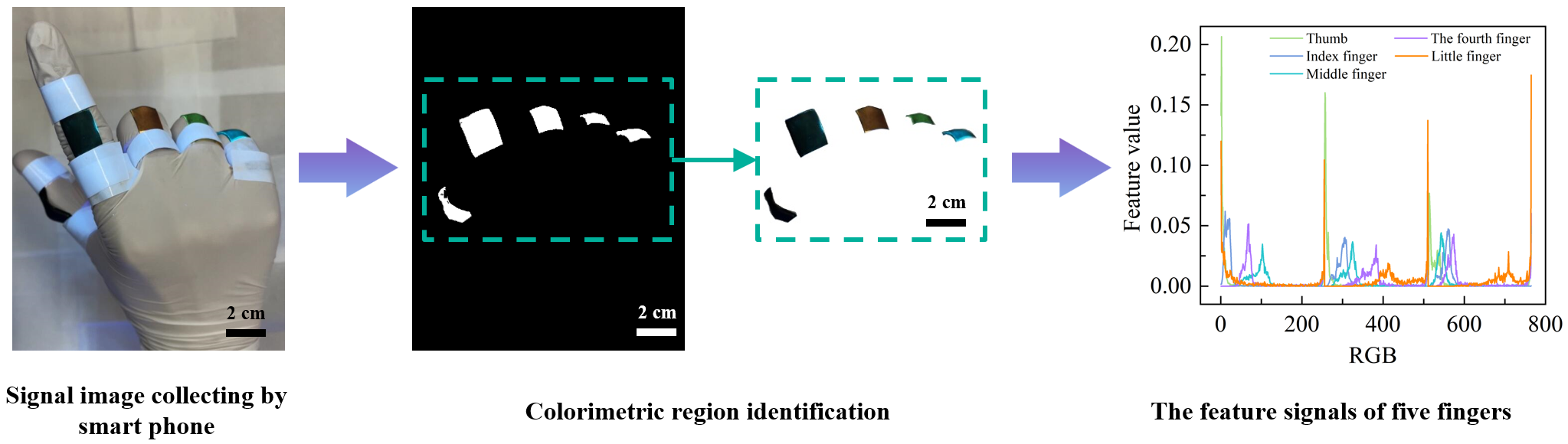


**Fig. S14** Process of the color data extraction and conversion to RGB distribution from the image of a human hand wearing the smart glove

**Table S6** Parameters of 5D-CNN-GRU model for hand gesture recognition

| Layer | Name | | Input  shape | Number of neurons | Return sequences | Activation function | Output shape |
| --- | --- | --- | --- | --- | --- | --- | --- |
| 1 | Input | Chanel 1 | 765×1 | － | － | － | 765×1 |
|  |  | Chanel 2 | 765×1 |  |  |  | 765×1 |
|  |  | Chanel 3 | 765×1 |  |  |  | 765×1 |
|  |  | Chanel 4 | 765×1 |  |  |  | 765×1 |
|  |  | Chanel 5 | 765×1 |  |  |  | 765×1 |
| 2 | Function model | | 765×1 | － | － | － | 753×64 |
|  |  |  | 765×1 |  |  |  | 753×64 |
|  |  |  | 765×1 |  |  |  | 753×64 |
|  |  |  | 765×1 |  |  |  | 753×64 |
|  |  |  | 765×1 |  |  |  | 753×64 |
| 3 | Concatenate | | 753×64 | － | － | － | 753×320 |
|  |  |  | 753×64 |  |  |  |  |
|  |  |  | 753×64 |  |  |  |  |
|  |  |  | 753×64 |  |  |  |  |
|  |  |  | 753×64 |  |  |  |  |
| 4 | GRU | | 753×320 | 64 | True | Linear | 753×64 |
| 5 | GRU | | 753×64 | 32 | False | Linear | 32 |
| 6 | Dense | | 32 | 1 | － | Softmax | 11 |

**Table S7** The network parameters of 5D-CNN-GRU Functional model

| Layer | Name | Input  shape | Number of neurons | Convolution kernel | Step size | Activation function | Output shape |
| --- | --- | --- | --- | --- | --- | --- | --- |
| 1 | InputLayer | 765×1 | － | － | － | － | 765×1 |
| 2 | Conv1D | 765×1 | 16 | (4,1) | 1 | Relu | 762×16 |
| 3 | Conv1D | 762×16 | 16 | (4,1) | 1 | Relu | 759×16 |
| 4 | MaxPooling1D | 759×16 | － | － | 1 | － | 759×16 |
| 5 | Conv1D | 759×16 | 64 | (4,1) | 1 | Relu | 756×64 |
| 6 | Conv1D | 756×64 | 64 | (4,1) | 1 | Relu | 753×64 |
| 7 | MaxPooling1D | 753×64 | － | － | 1 | － | 753×64 |
